# Pathways to Safety: A qualitative evaluation of an Australian domestic violence training program for primary care

**DOI:** 10.1186/s12875-026-03297-3

**Published:** 2026-04-01

**Authors:** Jacqueline Kuruppu, Fiona Giles, Simone Gleeson, Kitty Novy, Kelsey Hegarty

**Affiliations:** 1 Department of General Practice and Primary Care, The University of Melbourne, Parkville, Australia; 2 La Trobe Rural Health School, La Trobe University, Bendigo, Australia; 3 The Royal Women’s Hospital, Parkville, Australia

**Keywords:** Primary care, Domestic violence, Education, Training

## Abstract

**Background:**

General practitioners are among the most trusted professionals for disclosures of domestic violence (DV). However, primary care health professionals often perceive they lack the skills to appropriately respond. To address this need, the Pathways to Safety Training Program was delivered, aiming to increase readiness to respond to DV in primary care using a whole-of-practice approach. This qualitative study sought to evaluate the experiences and impact of the program on participants and program facilitators.

**Methods:**

Twenty-five participants from primary care clinics and facilitators of the training program were interviewed about their experiences of the program and its impact on their practice. Interviews were audio-recorded, transcribed verbatim and thematically analysed.

**Results:**

We generated five themes: *The collaborative*,* whole-of-practice approach engaged and motivated learners; Skilled and sensitive facilitators created safe spaces; Training encouraged professional and personal connection; Training elevated the response to DV*; *and Participants identified opportunities to expand the program*. A whole-of-practice approach led to opportunities for appreciation, interaction, and collaboration between colleagues. These opportunities were managed by skilled facilitators who were trained and supported to create safe spaces. This encouraged professional and personal connection which enhanced the spirit of collaboration post-training and provided the context for in-depth reflection on attitudes about DV. This, in conjunction with the practical skills learned during the training, led to an elevation of the response to DV within primary care. Participants identified opportunities to expand the program including follow-up training sessions to encourage accountability, more advanced training and an in-person delivery option.

**Conclusions:**

From a qualitative perspective, Pathways to Safety training program positively impacted the readiness of primary care to respond to DV. Two features of this training program are the whole-of-practice approach and co-facilitation by general practitioners and DV workers, which created opportunities for collaboration, reflection and conversation to improve the response to DV. We recommend the development and sustained funding of training programs which use this structure to enhance practice and service cohesion on sensitive clinical issues.

**Supplementary Information:**

The online version contains supplementary material available at 10.1186/s12875-026-03297-3.

## Background

Domestic violence (DV), including intimate partner violence, is a prevalent global public health issue, which disproportionally affects women, with one third of women experiencing physical or sexual violence by partners worldwide [1]. Women experiencing DV face damaging consequences to their physical and mental health [1], with intimate partner violence being a leading cause of morbidity and mortality for women of child-bearing age [2]. Given the health impact of DV, victim-survivors attend health services more frequently than the general population [1, 3] .

In Australia, general practitioners (GPs) are among the most trusted professionals for DV disclosures [4–7] and research shows that DV victim-survivors want to be asked about their situation in health settings [8]. Thus, primary care plays a key role in early intervention of DV. However, few families experiencing DV are recognised in health settings [9]. When disclosures do occur, the evidence suggests that health professionals may lack the skills to appropriately respond [5, 6, 10].

A 2021 Cochrane systematic review identified 19 trials of health professional focused training on intimate partner violence [11]. Three-quarters of these trials were set in the United States of America (USA) and there was a low to very low certainty for the achievement of outcomes from training [11]. Training impacted self-reported attitudes, knowledge and readiness, however it did not demonstrate impact on practitioners’ referral practice or the health and wellbeing of victim-survivors [11]. The authors of the review called for further research focusing on the impacts of DV training [11, 12] and training that is supported by system interventions.

There have been limited randomised trials of system interventions in primary care, which include more than simply training of health professionals [11, 12]. One such trial was the Women’s Evaluation of Abuse and Violence care (WEAVE) trial in Australia testing the effect of brief women-centred care counselling by trained GPs for women afraid of a partner/ex-partner [11, 13]. This study found that trained GPs enquired more about safety of the women and their children, and that depression outcomes were better for women who attended the counselling. WEAVE also showed that GPs could be trained to respond in a supportive, woman-centred way, and improve their knowledge, skills and attitudes [11]. Another trial conducted in the United Kingdom, IRIS (Identification and Referral to Improve Safety), testing training and an electronic prompt to ask about DV saw a marked increase in GPs’ identification of female victim-survivors and referral to specialist services [14]. An expansion of the program to recognize and refer men and children in DV contexts was shown to be acceptable and feasible [15].

### The Pathways to safety program context

**Fig. 1:**
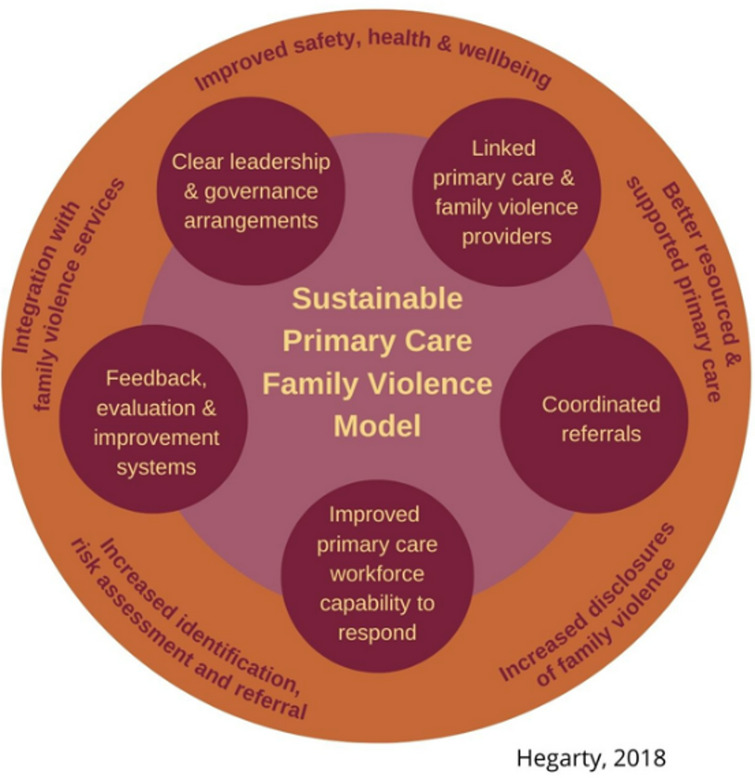
Sustainable primary care model

The Readiness Program was a national training program funded by the Australian Government from 2020 to 2024 to strengthen primary care’s capacity to identify and respond to domestic violence (DV) within a trauma and violence informed framework [16]. Based on the Sustainable Primary Care Model (see Fig. 1 and Table 1), education and referral capability are brought together to provide trust, create linkages and strengthen the relationships between primary care and domestic and family violence (DFV) sectors. The Pathways to Safety component of the training continues to be funded and delivered by the Safer Families Centre.

**Table 1:** Features underpinning the sustainable primary care model

Features underpinning the model are:
• Clear leadership stcructure and governance arrangements at the practice, operational program and national level to provide oversight and management of the program;
• Linking primary care providers with domestic and family violence sector to improve coordination of referrals and pathways to safety
• Enhanced workforce capability through increased organisational support, resourcing and primary care training; and
• Timely feedback, evaluation and improvement systems to ensure long-term program sustainability.

The Readiness Program’s core training, Pathways to Safety, originally developed in 2018 by the Safer Families Centre, University of Melbourne, promotes a ‘whole-of-practice’ approach to building capability, linkages and systems for integrated support across primary care and the DFV sector. The Pathways to Safety program was built on evidence from the voices of practitioners and victim-survivors, and the curriculum of the WEAVE trial [11, 13, 15]. It was also informed by the inclusion of all practice staff in the training and GP and DFV workers co-facilitating from the IRIS trial [15]. However, the Pathways to Safety program differed from the IRIS program, in that it was longer, clinical and non-clinical staff were trained together, there was content on motivational interviewing skills and non-directive goal setting, and reinforcement activities using a practice checklist and audit. The training aims to support and build upon GPs’, nurses’, and other practice staff members’:

- Active listening and responding skills to build trust with patients.
- Access to up-to-date evidence and resources in responding to DV.
- Skills to assess readiness for change and non-directive goal setting.
- Promotion of changes in the clinic to support addressing DV in practice.

The eight-hour program consists of: a practice readiness checklist; a clinical audit; self-paced online learning modules; suggested reading including RACGP White Book; and a state or territory specific handbook containing information on local DFV related services. Two 90-minute interactive online sessions are co-delivered by skilled GP educators and DFV educators. The first online training session is for clinical and non-clinical staff and uses practical exercises focusing on active listening, attitudinal change, trauma and violence informed care and system change in the clinic. The second session is for clinical staff and focuses on identification and response to DV using simulated role playing. The training uses evidence-based models and frameworks such as the World Health Organisation LIVES (Listen-Inquire-Validate-Enhance safety-Support) and Safer Families CARE (Choice and control-Action and advocacy-Recognition and understanding-Emotional connection) to structure the response elements of the program [17–19].

The facilitation of experiential learning through modelling respectful behaviors with participants is a key part of the curriculum. Participants are supported in an ongoing way through the offer of community of practice sessions and follow up with a DFV educator from local services. Facilitators are supported to deliver the training through ‘train the trainer’ sessions and a community of practice to workshop any arising issues [14]. The training program is usually completed by participants over approximately two months, with pre surveys before training and post- training surveys at 3 months after completion.

There has been limited qualitative evaluation of this type of training program, especially in the Australian setting [20]. There have been few studies exploring participant’s experiences and perceived impact from any similar multifaceted training program [21]. IRIS has been evaluated through interviews of GPs and nurses exploring barriers and facilitators to undertaking DV work [22], perceptions on the IRIS model [23], effect of COVID on the model [24] and factors that influenced implementation of IRIS [25].

The need to understand from primary care participants their experiences of training programs will allow recommendations for future educational programs [21]. The aim of this research was to evaluate the National Pathways to Safety program 2020–2024. This paper presents the qualitative component which sought to answer the following research questions:

- What were participants’ and facilitators’ experiences of the Pathways to Safety program?
- What was the impact of the Pathways to Safety program on participating primary care staff and facilitators?

The qualitative component was carried out by the first author who had a previous relationship with the Pathways to Safety team but was separate from the design and delivery of the training program. The term ‘participants’ will be used to refer to those who participated in the Pathways to Safety Program and undertook the training. The term ‘facilitators’ will refer to the trainers or those who facilitated the training. The term ‘research participants’ will be used to collectively refer to participants and facilitators who participated in this evaluation research.

## Methods

### Study design

We used individual semi-structured interviews to produce rich data that provided in-depth exploration of research participants’ experience of the training and its impact. This study was underpinned by an interpretivist paradigm [26]. An interpretivist approach acknowledges that research participants’ experiences and the resulting impact of the program are intersubjective, and that the knowledge gained from the program is influenced by previous knowledge, experience, social and professional setting, and attitudes and beliefs [26].

### Research participants and recruitment

Research participants were recruited via purposive sampling from two different contexts: Australian general practices that had undertaken the Pathways to Safety training (755 staff from 103 practices); and Australian GPs and FV educators who facilitated the training (23 GPs and 30 FV facilitators). A more detailed description of Readiness participant characteristics is available in the whole of Program evaluation report [27]. Primary care staff were eligible to participate if they had attended at least one of the two training sessions provided through the program. Facilitators were eligible to participate if they had co-facilitated at least two training sessions with primary care staff. Staff and facilitators who met these criteria were directly contacted by the research team via email with an information sheet to gauge interest in participating in an interview. Following expression of interest, the consent form was signed, and an interview time was arranged.

We deliberately selected 25 people to achieve diversity across a sampling frame in areas of professional background and levels of experience, gender, and practice location in order to capture different perspectives to generate rich and deep data. We recruited a broad range of participants from different professions and experience levels in the practice (i.e. GPs) and those from regional, rural and remote settings. The majority of participants were women (*n* = 24) which is reflective of the primary care staff and facilitators who participated in the program.

### Data collection

The interview guide for those who had participated in the training (participants) was focused on the following areas: motivation to undertake the training; experiences of the program including the impact of the training on their practice processes and clinic protocols; challenges and suggested improvements to the program; feedback on resources provided during the program; experiences with a family violence educator following the training and key factors for delivering this program to other general practices. Meanwhile, the interview guide for facilitators of the program explored the following points: experiences facilitating the program; aspects of the program that worked well; recommendations for change and improvement; what they believed participants gained from the program; the further training needs of participants; and any other reflections or comments.

Interviews were conducted by the first author between February 2024 and April 2024. The point at which interviews were conducted post-training differed for each participant. The average interview length was 24 min. All interviews were conducted over Zoom or on the phone as per research participants’ preference. The interviews were audio recorded with consent and were transcribed verbatim either by the first author or a commercial transcription agency. All transcripts were de-identified.

### Data analysis

Analysis was conducted by a multidisciplinary team of trauma-and-violence-informed women feminist researchers, with JK and FG bringing their experiences as qualitative researchers in primary care and SG and KN bringing their knowledge of the program delivery, and KH bringing her experience as a GP and primary care researcher into health systems response to DV.

Data was analysed using a combination of inductive thematic analysis and deductive thematic analysis [28, 29]. Inductive thematic analysis was guided by Braun and Clarke’s reflexive thematic analysis framework [28, 29] to generate themes that described the overall experience and impact of the Pathways to Safety Program. This framework comprises of six steps: transcription; data familiarisation; generating initial codes through complete coding; searching for themes; reviewing themes; defining and naming themes; and producing the report. The first author engaged in data familiarisation by re-reading the transcripts. Initial codes were generated, and these were discussed in fortnightly meetings with the second author. Preliminary themes were developed, and these were reviewed by the research team to further develop them into overarching themes. To deductively analyse the data, the Program’s learning objectives were used as a coding framework. Data was coded to the particular learning objective to which it was related. These findings were then combined with the preliminary themes from inductive thematic analysis to form the overarching themes. Overarching themes were discussed and revised by the whole team until consensus was reached that the themes were informative and facilitated the most meaningful interpretation of the data [29]. This paper presents findings produced from both inductive and deductive analysis.

Ethics approval was obtained from the Human Ethics Advisory Group at the University of Melbourne (Ethics ID: 25852).

## Results

Twenty primary care staff and five facilitators participated in the study. The majority of the research participants were women practicing in metropolitan settings (see Table 2). Participants’ and facilitators’ experience and the impact of the Pathways to Safety program was conceptualised across five themes: *the collaborative*,* whole-of-practice approach engaged and motivated learners*; *skilled and sensitive facilitators created safe spaces*; *training encouraged professional and personal connection; training elevated the primary care response to DV*; and participants *identified opportunities to expand the program.* Table 3 gives an overview of the subthemes relevant to each theme.

**Table 2:** Research participant demographics

Characteristic	Number	
	Participant	Facilitator
**Role in practice**		
GP	9	3
Nurse	4	N/A
**Practice Manger**	4	N/A
Administration staff	2	N/A
Allied health	1	N/A
Family violence educator	N/A	2
**State**		
NSW	6	2
SA	4	0
QLD	5	1
WA	3	0
VIC	0	1
TAS	0	1
NT	0	0
ACT	2	0
**Gender**		
Female	19	5
Male	1	0
**Area**		
Metro	12	1
Regional/Rural/Remote	5	4
**Age range** ^**a**^		
21 – 29	2	0
30 – 39	4	2
40 – 49	5	0
50+	6	3
**English as first language**		
Yes	19	5
No	1	0
**Country of birth** ^**a**^		
Australia	13	5
Other	6	0

^a^Data missing

**Table 3:** Themes and subthemes

Theme	Subthemes
The collaborative, whole-of-practice approach engaged and motivated learners (experience)	Practice interaction Feeling valued Collaborative opportunities
Skilled and sensitive facilitators created safe spaces (experience)	Facilitator relatability Management of sensitive conversations Supportive team environment
Training encouraged professional and personal connection (impact)	More frequent, in-depth reflection Developing a community of support Opening tea-room conversations
Training elevated the primary care response to DV (impact)	Individual level Practice level
Participants identified opportunities to extend the program (future)	Follow-up training Further advanced training In-person training option

### Theme 1: The collaborative, whole-of-practice approach engaged and motivated learners

General feedback from participants about the program was overwhelmingly positive and seemed to stem from the appreciation felt by staff that the training included the whole practice. For some, this was the main motivation for engaging in this training:

*I just thought it was important to have a holistic approach*,* you know from reception to me to everyone… I think that’s what motivated* [us to do the training] *the most.* Participant 17, Practice manager.

Whole-of-practice training had several benefits, the first being that it was an opportunity for staff to interact with their colleagues and see their practice methods:

*I think having it done as a full practice thing… you work with these people every day*,* but you never actually see them practice … It was useful how other people would react in that situation. I think it really helped.* Participant 16, GP.

Second, including practice staff who aren’t otherwise included in clinical training helped them to feel valued. One administration staff member reported feeling particularly valued when a doctor included the administration team in a strategy to improve the practice’s response to DV:

*One of our doctors during the session had a light bulb moment about how reception can be involved in alerting doctors to DV …which was* SO *helpful…that our doctors had a moment where they could go ‘oh*,* the admin team plays a role here.’* Participant 3, Practice manager.

Further, exposure to others’ roles within the practice created collaborative opportunities when designing clinic responses to DV:

*I think it’s really positive to allow the practices to attend as a whole because it kind of allows that whole of practice collaboration around this topic because it does involve more than just the GP.* Facilitator 1, GP.

While a whole-of-practice approach to training provided collaboration opportunities, there were some, both clinical and administration staff, who felt that parts of the training were not relevant to them. Perceived irrelevant content was met with minor frustration as they felt the training intruded on an already time-pressured environment.

*Because the first session was for all of the practice*,* so a couple of the reception staff did it. They did say they found it interesting*,* but they also said*,* I don’t know if it was really necessary for me.* Participant 8, Practice manager.

However, it was clear that for most participants, any potential irrelevance was outweighed by the gains in feeling valued, appreciation of different roles within the practice, and the opportunities for collaboration to establish a team approach to addressing DV.

### Theme 2: Skilled and sensitive facilitators created safe spaces

Opportunities for collaboration were managed by skilled facilitators’ ability to create safe spaces that encouraged change in the practice. All participants had positive reflections about the facilitators and particularly appreciated their relatability and management of sensitive conversations. Facilitators reported that these skills were supported and nourished by a supportive environment created by the Pathways to Safety team, presenting in pairs, and through opportunities to workshop issues arising from the training.

Facilitators’ relatability was a crucial factor in creating a safe space and was a powerful medium through which lessons from the training became accessible for participants:

*I just found they* [facilitators] *were very…engaging and relatable …certainly some of their experiences where they didn’t get it right too and missed something. …I just found it very practical …It makes it relatable…* Participant 17, Practice manager.

By being open and honest about their experiences in responding to DV, facilitators created a safe place for mistakes to be made and skills to be practiced within the session. This in turn seemed to make the idea of improving the response to DV achievable for participants.

The safe space created through relatability were further fostered through the skilled management of sensitive conversations. In understanding the prevalence of DV, facilitators were aware that survivors may be part of their audience. When participants decided to identify as survivors during the training, disclosures were met with sensitive management that provided an example to the clinic on how to respond to colleagues who were themselves victim-survivors of DV.

*Just the atmosphere of the teachers. Very compassionate… we hit a point which was quite sensitive*,* and I found out a bit about my work colleagues. So*,* I think we’re still a bit raw with emotions at the moment. But … it was a rewarding experience too because now we’ve been able to address other things and support one another. With having…the teachers there…when that was disclosed … they* [the facilitators] *could help us to work through it!* Participant 1, Practice manager.

Facilitators’ skill and presence seemed to be born not only of personal qualities but the training and support they themselves had received as facilitators in the program. All facilitators praised the Pathways to Safety Program administration team for their organisation and adaptability:

*I’ve really appreciated too how comprehensive the delivery package is and the support from the administration staff around arranging everything…because it really just helps you focus.* Facilitator 1, GP.

*It’s been a delight to work on this program…I think what makes it work so well*,* is that…everyone involved*,* …they’re all highly organised*,* they all can adapt…It’s always professional*,* but in a warm way. So*,* I have been very*,* very impressed… I can’t fault them.* Facilitator 2, FV educator.

The facilitators also found great value in presenting the training in pairs, where one brought their expertise on DV through their work as a FV educator, while the other was a GP and brought practical value to the presentation:

*The fundamental notion that we’re co-presenting with a content expert*,* I think it works really*,* really well. I think some slides*,* they’re just so much more credible when my psychosocial colleague delivers them… they can talk to that real world experience*,* and it really makes it more practical*,* more concrete that what we can do as GPs actually matters in the real world. So*,* I think that it was a stroke of genius to have decided to have the co-presenters.* Facilitator 3, GP.

Further supporting the facilitators were facilitator catch-ups using a community of practice approach, organised by the Pathways to Safety team, where the facilitators could bring their issues to workshop in a group environment.

*…we’ve had the support of the team around – I have never worked with a group that will have monthly facilitator meetings and have opportunities for people to ask questions.* Facilitator 2, DV educator.

In summary, the support and training facilitators received provided the foundation for the skills to create safe spaces within the training sessions. These safe spaces facilitated learning and provided an example of how to manage disclosures sensitively. In doing so, facilitators were creating the sparks for change within the practice, not only to better respond to patients experiencing DV, but to respond to colleagues who disclosed DV experience. The latter is further explored in the following theme.

### Theme 3: Training encouraged professional and personal connection

Following the training, some participants shared that the dynamics within the practice had changed, especially when a disclosure by a staff member of DV had been made during the training. This led to closer professional relationships between colleagues which were expressed in different ways. One of these ways was through more frequent and in-depth reflective discussions especially in small practices:

*Since finishing* [the training], *the nurse and I… probably talk about a little bit more in those discussions*,* we use each other as a soundboard … We…were doing it before but maybe not as in depth after the training.* Participant 6, Administration staff.

In larger practices, the training had opened tea-room conversations about DV and seemed to develop a community of support within the practice:

*… It helped us start conversations in the practice a bit more.* Participant 3, Practice manager.

Opening tea-room conversations also provided participants opportunities to understand and reflect on their and their colleagues’ attitudes towards DV and instigate change:

*It was interesting and it opened up a lot of tea-room conversations about this…One of the clinicians …ended up disclosing to me that she’d been a child of a domestic violence household and…her mother stayed and left her in that situation*,* and that’s why she…finds it hard when women don’t leave… I found that quite helpful for me to know that that’s their background*,* whereas before we never would’ve discussed that. …Then*,* after talking it through and having the training*,* I think they then got a different perspective*,* which really was helpful for them.* Participant 11, GP.

In the same way that the training empowered participants to start conversations, it also empowered them to apply the skills they had learned to support their colleagues experiencing DV:

[The training]*…opened up a lot more support for my work colleagues… it was quite empowering for us to be able to help each other and now we know how to support one another.* Participant 1, Practice manager.

It is important to note that not all participants experienced a greater professional and personal connection after the training. Some attributed this to the practice already having a sufficient relationship to reap the benefits of professional and personal connection before the training:

*I mean our practice…we do actually all work very closely together…We collaborate really well between each other.* Participant 8, Practice manager.

Meanwhile, for others, it appeared that not enough of the practice could attend the training to create the professional and personal connection effect:

*But then*,* we’ve only got that small population to work on*,* because it was only the three of us that went.* Participant 13, Nurse.

Overall, while only experienced by some, a powerful outcome of the training was encouraging greater professional and personal connection. The support this offered not only resulted in practice staff workshopping ideas to help respond to a patient’s or colleague’s situation, but it also empowered practice staff to change their attitudes and respond to colleagues’ experiences of DV. In this way, professional and personal connection was a factor for elevating practices’ responses to DV.

### Theme 4: Training elevated the primary care response to DV

Different people gained varying skills and knowledge from the training, with the majority gaining at least one benefit that elevated their response to DV. The impact from the training was experienced across individual and practice levels. At the individual level, participants gained tools to increase their comfort and confidence in approaching DV while change at the practice level seemed to occur on a continuum.

The tools participants gained at an individual level began with awareness of how to respond:

*I think the people who did that program*,* we all said the similar thing that*,* oh*,* it’s really helped me have that awareness around what to do. It* [the program] *certainly increased that knowledge around how to deal with…a domestic violence situation that’s brought up.* Participant 8, Practice manager.

Having an awareness about how to respond seemed to stem from a key message reiterated in the training: ‘you do not need to fix the problem’. This statement seemed to free participants from the perceived responsibility to end DV in one consult, which gave them permission to be more open to asking.

*Probably less worried about being in the position because … I’m not being called upon to try and fix the problem*,* I’m just being called upon to be supportive.* Participant 6, Administration staff.

In feeling more prepared to be aware and respond to DV, participants felt more comfortable and open to approach the issue of DV with their patients, which resulted in increased asking about DV:

*I feel like now I ask the question…it’s not just if you feel physically unsafe*,* if you feel like someone’s coercing you or people have been asking you for money. Wording it in different ways for different things*,* different people.* Participant 5, Nurse.

This approach, combined with clear strategies to elicit and address disclosures seemed to instill confidence in participants and their response to DV.

*I think I came away with some takeaways that really support me in addressing suspected domestic concerns. I think that that’s probably a big hurdle for a lot of healthcare workers*,* is that confidence.* Participant 15, Nurse.

Increased openness, strategies and confidence seemed to lead to a more sensitive response following the training, a skill that was transferrable to contexts beyond DV:

*I think it was good just knowing that it’s okay just to provide ongoing contact and open communication with the ladies instead of trying to fix it for them at that first disclosure…just that continuing communication around these issues is enough for some women.* Participant 11, GP.

*It’s definitely more taking more notice of patients that are walking in…There’s a few patients since then*,* if they come out quite upset*,* I lean forward*,* between and I go ‘are you okay to drive? Do you want to sit down for a bit first?’* Participant 10, Administration staff.

On a practice level, most participants demonstrated that they had assessed current clinic protocols and resources, either formally or informally and identified areas for improvement. However, following the training, change seemed to occur on a continuum. Some practices had discussed potential changes during the training, but were yet to enact them:

*That was one of our actions that came out of the training was that we were – we thought it would be a good idea for us to try and actually link in with local services… But again*,* has not yet eventuated.* Participant 18, GP.

Others had implemented some basic changes such as making resources visible in the clinic:

*As a whole*,* I know we got some little cards to put up*,* or put in the toilets I think*,* and things like that…Just little things that women or people can pop in their handbag or remember.* Participant 5, Nurse.

Some had done extensive research on local DV programs and gathered resources to be displayed in clinics:

*We researched a little bit and I reached out to some different programs…*,* nationally and statewide*,* and we got a few posters sent out to us and some pamphlets…We called one of the women’s health workers [nearby] and they sent us through a big list of all women’s shelters and women’s support groups…that they offered so that we could put that up in the clinic*. Participant 6, Administration staff.

Others changed the way they taught incoming medical students in light of what they had learned during the training:

*I train medical students. I think I put more emphasis after that course as well on getting the students to recognize…current symptoms…that could be signs of past trauma. I’ve definitely spent a bit more time on that.* Participant 14, GP.

Though, some did not implement any changes as their existing pathway was meeting their needs:

*…although it didn’t ever end up putting anything formal in place in terms of policies or anything…Because we actually having a social worker here…in this practice…who can then do the calling around and finding services and that sort of thing.* Participant 12, GP.

While Participant 12’s practice did not feel the need to change their policies, the learnings from the training culminated in an improved whole practice response to an acute instance of DV:

*Maybe a couple of months after we’d done the training – we had a fairly dramatic incident where a patient…had presented to the practice quite scared for her life… The partner turned up*,* the police were called…But the reception staff were then trained and just reacted perfectly… as the clinical director I got feedback that it was done incredibly well and the practice nurse said she was very impressed…our social worker sent an email around to everybody just congratulating the reception staff on their work.* Participant 12, GP.

The above quote is an example of how whole-of-practice training developed the skill and capability to respond to a high risk DV event in a safe and sensitive manner.

Whether through increased awareness, more tools for asking, being instilled with the message ‘you don’t have to fix the problem’, increased comfort and confidence to broach and address DV, or changes within the practice, the Pathways to Safety training improved awareness, behaviour and responses to DV at an individual and practice level in primary care.

### Theme 5: Participants identified opportunities to expand the program

While participants struggled to think of improvements to the training, there were some suggestions for the expansion of the program. These suggestions pertained to follow-up sessions with facilitators, more advanced training, and options for training format.

To enhance the training, participants asked for follow-up training sessions to hold the practice accountable to their commitment to improve their response to DV:

*I wonder whether*,* yeah*,* maybe having a six-monthly follow-up as part of the process would be good… To make us a bit more accountable… I still want to do more in this space.* Participant 11, GP.

There was feedback, too, from both participants and facilitators about the level at which the training was pitched. While the Pathways to Safety Program was advertised as introductory training for the response to DV, it also attracted practices that were more advanced in their knowledge. Thus, while the training provided reassurance for their current practices, there was also a call for training in more advanced skills, including engaging perpetrators or responding to DV in same sex relationships.

*The only other thing I think which might be handy…would be addressing domestic violence from both perspectives. Because when you see a perpetrator and victim*,* or when both parties are intermittently perpetrator and victim…it’s a really tricky topic… Maybe some training addressing the perpetrators as well and bringing that up as a topic.* Participant 16, GP.

*We probably need a very*,* very short bespoke program on same sex abuse for practices that are in areas where there’s a lot of same sex-oriented people in their practice.* Facilitator 3, GP.

Further, participants and facilitators alike expressed the desire for a face-to-face option for the training. It was felt that this format would be more engaging:

*I really love in person training sessions and I find them super valuable… it just makes it feel like it’s more intentional and more aimed at you and like you’re more involved in it.* Participant 3, Practice Manager.

For facilitators, an in-person session would allow them to monitor non-verbal cues and adjust their delivery accordingly.

*I can see value to having a face-to-face option… People would have their cameras turned off so I can’t look for interpersonal cues of when to go*,* when to move on.* Facilitator 5, Family violence educator.

In summary, participants and facilitators felt that the Pathways to Safety Program could be extended by offering follow-up training and sessions on advanced skills and by offering an in-person option for practices to enhance engagement.

## Discussion

Research participants’ experiences of the Pathways to Safety training program and its individual and practice level impact was conceptualized through five themes: *The collaborative*,* whole-of-practice approach engaged and motivated learners; skilled and sensitive facilitators created safe spaces; Training encouraged professional and personal connection; Training elevating the response to DV; and Participants identified opportunities to expand the program*. A key point of difference between this program and other programs was the whole-of-practice approach of Pathways to Safety [21]. A whole-of-practice approach led to opportunities for appreciation, interaction, and collaboration between colleagues. These opportunities were managed by skilled GP and DFV facilitators who were trained and supported to create safe spaces within the training. These safe spaces encouraged professional and personal connection which enhanced the spirit of collaboration post-training and provided the context for in-depth reflection on attitudes and practice about DV. This, in conjunction with the practical skills learned during the training, led to an elevation of the response to DV within primary care. Research participants identified opportunities to expand the program including follow-up training sessions to encourage accountability, advanced training and an in-person delivery option.

The interpretive approach of this research acknowledged that the gains from the training program were influenced by context and a participants’ readiness to undertake this sensitive work. Those who spoke about benefiting most from the training were those who had little previous knowledge of DV. Other relevant contexts that facilitated positive impacts included the program reaching a critical mass of staff within the practice, and practices with lower levels of professional connection before engaging with the program. However, we know that there are many factors that help a health professional become ready to do this work. These include a personal commitment, having an advocacy approach, trusting that health settings are a good place to do this work and collaborating with a team [5]. It is likely that the staff who participated in this program inherently possessed these qualities, which may have facilitated the positive impacts presented in this paper. Some of the most powerful impacts of the training were collaboration, feeling valued and professional and personal connection at the practice level.

Relational leadership theory may have had role in producing impacts of collaboration, feeling valued and professional and personal connection. Relational leadership is a leadership theory that builds social influence through relationships, rather than positions of power or individual traits [30]. The program introduced a relational approach to clinical operations through its whole-of-practice approach which encouraged inclusivity and empowerment of all staff, which are core principles of relational leadership theory [30]. This strength of the program may be key to achieving the impacts of collaboration, safe spaces and connection.

These impacts may also stem from beyond the written curriculum and perhaps from a hidden curriculum. Hidden curriculum refers to the implied norms, values, and beliefs gained through the underlying structure and social aspect of the training [31]. This included training facilitators to model respectful behaviours with the training participants in a student-centred way to enable them to practice in a patient-centred way [13]. Further, participants disclosing DV and the subsequent management of those disclosures by facilitators provided implicit education through behavioral modelling [32] on managing both disclosures and workplace relationships. However, not all participants experienced an increase in professional and personal connection, either because staff were already connected or too few practice staff attended or engaged in discussions post-training to change the dynamics of the practice.

### Strengths and limitations

This qualitative evaluation was limited by several factors. First, the interviews were not conducted at a set time-point following the training. This meant that some interviews were conducted shortly after the training where not enough time had passed to assess any changes; or in other cases the interview was conducted well after the training resulting in poor memory. Thus, there may be elements of the impact of the training that have not been perceived. Second, the findings of this evaluation are based on those who self-selected into the study and are subject to participation bias. While no one we invited into the study actively declined, several potential participants did not respond to our invitation. We do not know if their views would be different from those we did interview. Additionally, the findings are generated from self-reported responses, meaning the impact of the training may be over or understated. Further, patient perspectives were not included in this study, making it difficult to assess the impact of the program on patient experiences of responses to DV in primary care. Third, not every state and territory were represented in the sample, meaning that differences in perspectives due to practicing and applying learnings in different locations, in context of differing policy frameworks and specialist service support systems may not have been captured.

Nevertheless, there were a diverse range of professions in the sample to reflect the inclusion of the whole-of-practice in the study. Another strength is the inclusion of facilitators as they were able to provide broader feedback about the program and reflect not just their own views but their perspectives of others’ experiences which added more depth to the analysis.

### Implications

The Pathways to Safety whole of practice training program delivered by co-facilitators from GP and DFV sectors was appropriate for primary care staff to upskill in addressing DV. This program needs to be further evaluated to ensure feasibility and effectiveness. Further evaluation should include an outcomes evaluation based on patient experiences of disclosure in primary care settings. The Pathways to Safety training program model may also have implications for other contexts, including responding to social determinants of health including social isolation and food security, to which the skills taught in this program may be transferred.

## Conclusions

From a qualitative perspective, the Pathways to Safety program positively impacted the readiness of primary care to identify and respond to DV. The program achieved a greater awareness of DV prevalence and provided general primary care staff with a framework for the mindset and tools to respond to DV. The major features of this training program was that it was a whole-of-practice initiative and it was jointly facilitated by GPs and specialist DFV Workers. This approach created opportunities for collaboration, reflection and conversation designed to improve the response to DV in practice. We recommend the development and sustained funding of training programs which use this structure to enhance practice and service cohesion on sensitive clinical issues.

## Supplementary Information

Supplementary Material 1.

Supplementary Material 2.

## Data Availability

The authors declare that data supporting the findings of this study are available within the article.

## Abbreviations

DV: Domestic Violence
DFV: Domestic and Family Violence
FV: Family Violence
GP: General Practitioner
NSW: New South Wales
SA: South Australia
QLD: Queensland
WA: Western Australia
VIC: Victoria
TAS: Tasmania
NT: Northern Territory
ACT: Australian Capital Territory
