# Supplementary material for: Pathways to Safety: A qualitative evaluation of an Australian domestic violence training program for primary care
Source: BMC Prim Care. 2026 Apr 1;27:188. doi: 10.1186/s12875-026-03297-3 (PMC13169565; doi:10.1186/s12875-026-03297-3)
Supplement: Supplementary file 1 — Supplementary Material 1. [file 12875_2026_3297_MOESM1_ESM.docx]

# Pathways to Safety Evaluation: Interviews

## Interview Guide: training participants

**Opening**

- Open with verbal consent script
  (as per document: ‘Verbal consent script_Pathways to Safety_Interviews_V1_24042023’)
- If consent is provided continue with interview as per guide below.

**Interview Guide: Topics/questions**

***Training***

1. What motivated you to do the training program?
2. Tell me more about your experiences with the program….
   1. How did you feel about doing the training program?
3. Were there any outcomes or benefits of the program for you?
   1. What things did you take on board in your practice? Did the training have any impact on your practice?
   2. How emotionally ready are you to respond to family violence following the training?
4. Were there any challenges of the program for you?
5. Can I ask you some specific questions – how did you find the:

- Handbook?
- Modules e-Learning?

1. What could we improve in the program?

***Family violence worker – after the training contacts with specialist service***

1. What was your experience with follow ups and engagement with the Family Violence worker?
   1. Has this had any impact on your practice?
   2. How could this be improved?

***Overall***

1. What are key factors for any roll out of this program to other practices?
2. Are there any other comments you would like to make?

**Close**

Thank participant for their time.
