# Supplementary material for: Pathways to Safety: A qualitative evaluation of an Australian domestic violence training program for primary care
Source: BMC Prim Care. 2026 Apr 1;27:188. doi: 10.1186/s12875-026-03297-3 (PMC13169565; doi:10.1186/s12875-026-03297-3)
Supplement: Supplementary file 2 — Supplementary Material 2. [file 12875_2026_3297_MOESM2_ESM.docx]

# Pathways to Safety Evaluation: Interviews

## Interview Guide: Trainers (GP Facilitators and FV Support Workers)

**Opening**

- Open with verbal consent script
  (as per document: ‘Verbal consent script_Pathways to Safety_Interviews_V1_24042023’)
- If consent is provided continue with interview as per guide below.

**Interview Guide: Questions**

1. Can you tell me about your experiences of the Pathways to Safety Training Program

2. What aspects of the Program are working well?

3. What aspect of the Program would you change?

4. What do you think participants gain from the Program?

5. What else do you think participants would want training in?

6. How well do you think the training addresses emotional readiness?

6. Do you have any other reflections of the Program?

7. Are there any other comments you would like to make?

**Close**

Thank participant for their time.
